# A Combined Ray Tracing Method for Improving the Precision of the USBL Positioning System in Smart Ocean

**DOI:** 10.3390/s18103586

**Published:** 2018-10-22

**Authors:** Jian Li, Qi Gu, Ying Chen, Guiqing Sun, Haocai Huang

**Affiliations:** 1 Department of IOT Engineering, Hohai University, Changzhou 213022, China; jian263@sina.com (J.L.); noyaqq@163.com (Q.G.); 2 State Key Laboratory of Acoustics, Institute of Acoustics, Chinese Academy of Sciences, Beijing 100190, China; 3 Ocean College, Zhejiang University, Zhoushan 316021, China; ychen@zju.edu.cn (Y.C.); sgq@zju.edu.cn (G.S.)

**Keywords:** acoustic positioning, constant sound speed ray tracing, equal gradient ray tracing, sound speed profile, ultra-short baseline (USBL)

## Abstract

The ultra-short baseline positioning system (USBL) has the advantages of flexible application and easy installation, and it plays an extremely important role in the underwater positioning and communication. The error of the USBL in underwater positioning is mainly caused by a ranging error due to ray tracing, a phase difference error of the USBL, and acoustic noise in the underwater communication. Most of these errors are related to the changes in the sound speed during its propagation through the ocean. Therefore, when using the USBL for underwater detection, it is necessary to correct the sound speed profile in the detection area and optimize the ray tracing. Taking into account the actual conditions, this paper aims at correcting the model of underwater sound speed propagation and improving the tracking method of sound lines when the marine environment in the shallow sea area changes. This paper proposes a combined ray tracing method that can adaptively determine whether to use the constant sound speed ray tracing method or the equal gradient ray tracing method. The theoretical analysis and simulation results show that the proposed method can effectively reduce the error of slant distance in USBL compared with the traditional acoustic tracking method and the constant sound speed ray tracing method. The proposed sound ray correction algorithm solves the contradiction between the number of iterations and the reduction of positioning error and has engineering application value.

## 1. Introduction

The ultra-short baseline positioning system (USBL) is based on the installation of an ultra-short baseline array on a ship bottom and a transponder mounted on an underwater target. The transmitter emits an acoustic signal, and the underwater target receives the transmitted acoustic signal and sends back an answer signal. After receiving the signal, the USBL calculates the azimuth and distance [1] of the target using the time-delay difference or phase difference between each receiving hydrophone [2], and calculates the position coordinates of the underwater target. The ultra-short baseline positioning system (USBL) is generally used for underwater positioning in shallow sea area [1].

Due to the complexity of the actual marine environment, the accuracy of an ultra-short baseline positioning system is limited mainly due to the existence of the ranging error, phase difference error, transmission and reception delay estimation error, and acoustic noise in the communication. The main reason for the above errors is as follows. When an acoustic signal propagates underwater, the sound speed is affected by the influence of the marine environment (such as temperature, depth of the seabed, salinity, etc.). Namely, the sound ray will propagate along a curved trajectory, which aggravates the prediction of the ray propagation paths such as refraction and reflection, making it difficult to implement the sound-tracking and compensation measures effectively. The acoustic propagation path bending caused by the sound speed profile affects the accuracy of distance measurement in the underwater acoustic propagation and causes errors which make a deviation from the true value large [3]. Therefore, it is necessary to optimize the sound speed profile in the shallow sea area and to compensate the sound ray in the underwater acoustic propagation, to improve the positioning accuracy of the ultra-short baseline.

At present, there are many international studies on the methods of correcting the model of sound speed profile such as the empirical sound velocity [4], weighted average sound velocity method [5], polynomial fitting method [6,7], the method of equivalent speed profile [8], effective speed of sound see table and look-up table method [9,10], etc. The above methods are based on the original sound velocity data. Although these methods can simulate the sound speed profile well, they abandon the measured sound velocity profile data completely, which easily leads to a poor sound speed correction in the shallow water areas. However, it is difficult to provide the effective data of sound speed proflie in complex shallow water areas. Therefore, in order to accurately restore the sound speed profile in the actual environment and improve the ray tracing method, this paper uses an empirical orthogonal function (EOF) to invert the sound speed profile [11]. This paper proposes a combined ray tracing method that can be used in the USBL to adaptively determine whether to use the constant sound speed ray tracing method or the equal gradient ray tracing method. The simulation experiments show that the proposed method has better flexibility than the traditional ray tracing and positioning method, and the positioning accuracy is improved keeping the amount of calculation iterations basically the same. It should be claimed that, in this work, the amount of iterations is not same as the amount of calculation. If the work done in each iteration is different between the compared processes, the amount of calculation compared between several methods could be different.

This paper is divided into eight parts. Section 1 describes the principle of ultra-short baseline positioning optimization and the reasons for the positioning error, and briefly introduces the existing technology of sound ray tracing method. Section 2 introduces the specific methods and principles of ultra-short baseline positioning. Section 3 introduces the principle method of empirical orthogonal function. Section 4 introduces the basic theory of ray acoustics in layered media. Section 5 introduces two ray tracing methods: constant sound speed ray tracing method and equal gradient ray tracing method. Section 6 proposes a combined ray tracing method applied in ultra-short baseline. Section 7 introduces the original traditional sound ray tracing model and the principle of slant distance correction after using ray tracing methods. Section 8 is the summary of simulation experiments and Section 9 is the summary and outlook.

## 2. USBL Positioning Principle

The USBL achieves the underwater target positioning by transmitting and receiving acoustic signals [12]. The USBL consists of at least three hydrophones and a transmitter placed on a ship bottom. After a transmitter emits an acoustic signal, the transponder placed on the underwater target receives the transmitted signal. According to the time that passes from transmitting to receiving of the underwater acoustic signal, the distance between the target and an ultra-short baseline array can be obtained. The azimuth of the underwater target to the transceiver head is calculated based on the time difference or phase difference of arrival of the acoustic wave at each hydrophone, and the three-dimensional position coordinates of the underwater target are obtained through the geometric formula [13]. 

**Figure 1:**
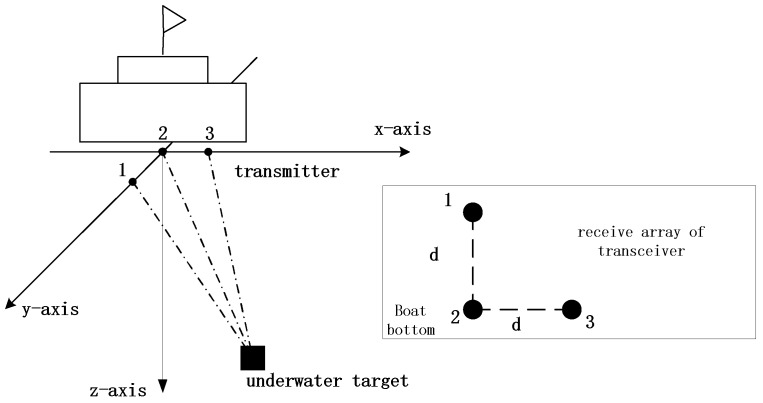
Ultra-short baseline positioning principle.

As shown in Figure 1, the ultra-short baseline adopts a right-angled triangle array with a spacing of *d*. The right-angle triangle model is simple, streamlined, easy to calculate, and quick to understand. Let set No. 2 hydrophone as an array center (0, 0, 0), then the phase difference [14] between the baseline and the other two hydrophones is $\psi_{12}$ and $\psi_{22}$ respectively. The obtained sound speed is converted to the wavelength using the relation

(1) c=f⋅λ

where f is the center frequency of the received signal, c is the speed of the sound, and *λ* is the wavelength corresponding to the frequency f; all quantities are measured by the underwater acoustic monitoring equipment. Using the USBL principle and geometry physics, the three-dimensional coordinates of the target are

(2) $$l^{2}=X^{2}+Y^{2}+H^{2}$$

(3) $$\psi_{12}=\frac{2\pi }{\lambda }\left[\sqrt{l^{2}}-\sqrt{{\left(d-X\right)}^{2}+d^{2}+H^{2}}\right]$$

(4) $$\psi_{22}=\frac{2\pi }{\lambda }\left[\sqrt{l^{2}}-\sqrt{X^{2}+{\left(d-y\right)}^{2}+H^{2}}\right]$$

where *d* is the spacing between the array elements, $\psi_{12}$ and $\psi_{22}$ are the phase difference between the second and the other two hydrophones, and H is the height of the target array element, X is the distance of the target along the x-axis direction, Y is the distance of the target along the y-axis direction, l is the slant distance of the target from the USBL, and triple (X, Y, H) denotes the three-dimensional coordinates of the target relative to the array.

Sound propagation speed has a very little effect on the phase difference of the signals received by the hydrophones in the transceiver head. One of the main factor affecting the positioning accuracy of the USBL is the measuring error caused by sound propagation in the slant range. Therefore, it is particularly necessary to take into account the form of the sound propagation path to improve the slant range accuracy of the target and the array.

## 3. Empirical Orthogonal Function

The sound underwater propagation path and attenuation are closely related to the change of underwater sound speed. The change of the sound speed profile (SSP) is one of the important factors affecting the sound propagation in the ocean [15]. The sound speed profile is a structural distribution of the path and velocity variations of an acoustic wave propagating in the seawater. Due to the marine environment misalignment caused by the environmental factors such as the non-uniformity of seawater medium, and acoustic propagation channel interference [16], climate change [17,18,19], and other various reasons such as failure of the equipment for sound speed measuring, the sound speed profile measured in real time has diversity and errors. The empirical orthogonal function is a very effective method for inversion of the sound velocity profile. The EOF can obtain accurate enough sound speed profiles with the history sound speed data. The research of Davis et al. [20] showed that the order of EOF is associated with the ocean physics processes, and usually, only the first few orders are needed to represent the sound speed profile accurately.

The EOF utilizes the spatiotemporal correlation of sound velocity profile data. After feature vector extraction and modal decomposition of the group of the sound speed profiles with the same characteristics, a common feature vector is obtained and combined with the sampled data, so that an accurate representation of the continuous sound velocity profile data can be achieved [21].

Assuming there are *M* measured sound velocity profiles in the measurement area, *M* sound speed profile samples can be obtained, which are expressed as

$$c_{1}(z_{i}),c_{2}(z_{i}),\cdots ,c_{M}(z_{i})\;i=1,2,\cdots N$$

where *N* is the number of discrete points in the depth direction. These samples can be expressed as [22]

(5) $$C=\left[\begin{array}{l} c_{1}(z_{1})\;c_{2}(z_{1})\;\cdots \;c_{M}(z_{1})\\ c_{1}(z_{2})\;c_{2}(z_{2})\;\cdots \;c_{M}(z_{2})\\ \;\vdots \;\vdots \;\vdots \;\vdots \\ c_{1}(z_{N})\;c_{2}(z_{N})\;\cdots \;c_{M}(z_{N}) \end{array}\right]$$

The average of *M* speed profiles is obtained by

(6) $$\bar{c}=\frac{1}{M}\sum_{j=1}^{M}c_{j}(z_{i})=\left[\begin{array}{l} \bar{c}(z_{1})\\ \bar{c}(z_{2})\\ \vdots \\ \bar{c}(z_{N}) \end{array}\right]$$

In the sound speed matrix *C*, *M* sound speed profiles are subtracted from the average sound speed profile $\bar{c}$ respectively to obtain the perturbation of each sound speed profile with respect to the average sound speed profile; namely, the perturbation matrix ΔC is defined as

(7) $$\Delta C=\left[\begin{array}{l} \Delta c_{1}(z_{1})\;\Delta c_{2}(z_{1})\;\cdots \;\Delta c_{M}(z_{1})\\ \Delta c_{1}(z_{2})\;\Delta c_{2}(z_{2})\;\cdots \;\Delta c_{M}(z_{2})\\ \;\vdots \;\vdots \;\vdots \;\vdots \\ \Delta c_{1}(z_{N})\;\Delta c_{2}(z_{N})\;\cdots \;\Delta c_{M}(z_{N}) \end{array}\right]\;$$

where $\Delta c_{j}(z_{i})=c_{j}{(z}_{i})-{\bar{c}(z}_{i})\;i=1,2,\cdots ,N,\;j=1,2,\cdots ,M$. The covariance matrix *R* of the sound speed perturbation matrix is defined by

(8) $$R=\frac{\Delta C\Delta C^{T}}{M}$$

The covariance matrix *R* represents the uncertainty of the fluctuation in the sound speed in the measurement area. The characteristic decomposition of *R* results in

(9) RF=DF

In Equation (9), $D=[\lambda_{1}\lambda_{2}\cdots \lambda_{N}]$ is the eigenvalue matrix of the covariance matrix *R*, and *F* is the matrix of the eigenvectors of *R*, expressed as

(10) $$F={\left[f_{1}(z)\;f_{2}(z)\;\cdots \;f_{N}(z)\right]}^{T}$$

The component $f_{i}(z)$ corresponding to the eigenvalue $\lambda_{i}$ is the ith EOF mode.

The above *N* feature values $\lambda_{1},\lambda_{2},\ldots ,\lambda_{N}$ are arranged in ascending order. The sound velocity profile at any point in the measurement region can be represented by the first *K*-order EOF approximation as

(11) $$c(z)=\bar{c}+\sum_{i=1}^{K}\alpha_{i}f_{i}(z)$$

In Equation (11), $\alpha_{i}$ is the EOF coefficient of the sound speed profile. The determination method of $\alpha_{i}$ is

(12) $$\alpha_{i}={\left[\alpha_{1}\;\alpha_{1}\;\cdots \alpha_{i}(K)\right]}^{T}$$

where ${\left[\alpha_{1}\;\alpha_{2}\;\cdots \alpha_{i}\;\cdots \;\alpha_{M}\right]}_{K\times M}=A_{K\times M}$, $A_{K\times M}=\frac{\Delta C}{F_{N\times K}}$.

Equation (11) can be used to represent the values of each sound velocity profile retrieved in the SSP. Normally, the sound velocity profile can be well inverted by selecting 3 to 6 orders of the EOF [23].

## 4. Basic Theory of Ray Acoustics in Layered Media

**Figure 2:**
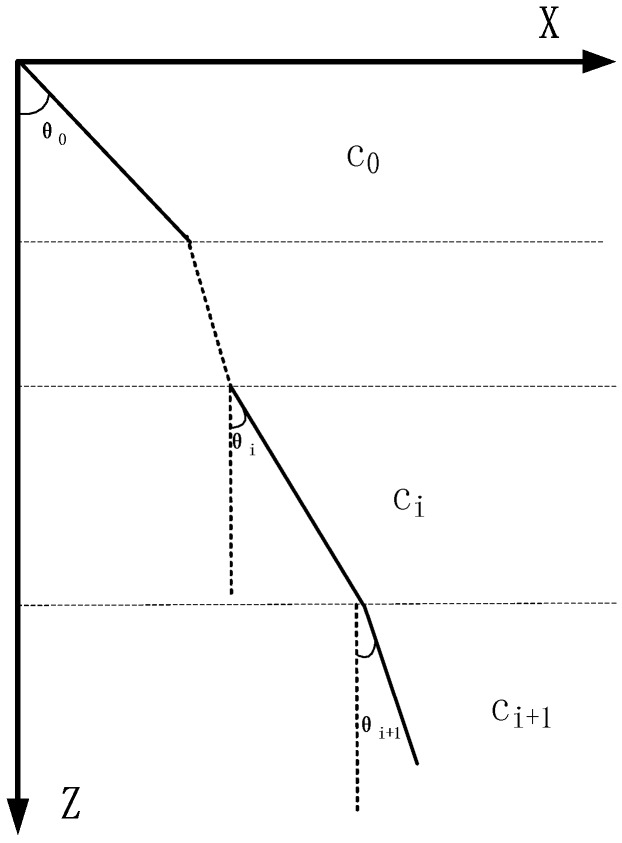
Sound transmission in the water.

Ray acoustic is an intuitive sound field analysis method, which has the good robustness to the complex media and boundary inversion. Moreover, it is an important method to analyze the sound field. The basic assumption of the ray acoustics is that acoustic waves are transmitted along a certain direction, and the trajectory of acoustic waves is the sound line, and the ray lines are perpendicular to the isosceles (wavefronts) [24]. As illustrated in Figure 2, in a layered media (where there is a border between two media or the properties of the same media are changed), the acoustic wave will be reflected and refracted, and the propagation path of acoustic wave will always bend toward the area with a lower sound speed. The propagation law of sound lines cross over the medium satisfies the Snell’s law, which is expressed as

(13) $$\frac{\sin\theta_{i}}{c_{i}}=\frac{\sin\theta_{i+1}}{c_{i+1}}=p$$

In Equation (13), $\theta_{i}$ is the angle between the sound wave of the *i*th layer and the normal to the horizontal plane, and it is called the sonic incident angle of the *i*th layer, $c_{i}$ is the sound speed of the *i*th layer, and *p* is the Snell constant.

In different layers of a propagation media, the sound speed changes, so the propagation path of the sound is not a straight line [25]. Using the Snell’s law, we can develop a method for determining the sound path. Presently, two methods are commonly used for acoustic path tracking [26], the constant sound speed ray tracing method, where it is assumed that the sound speed in the layer is constant, and the equal gradient ray tracing, where it is assumed that there is a gradient change in the sound speed in the layer.

## 5. Ray Tracing Methods

### 5.1. Constant Sound Speed Ray Tracing Method

**Figure 3:**
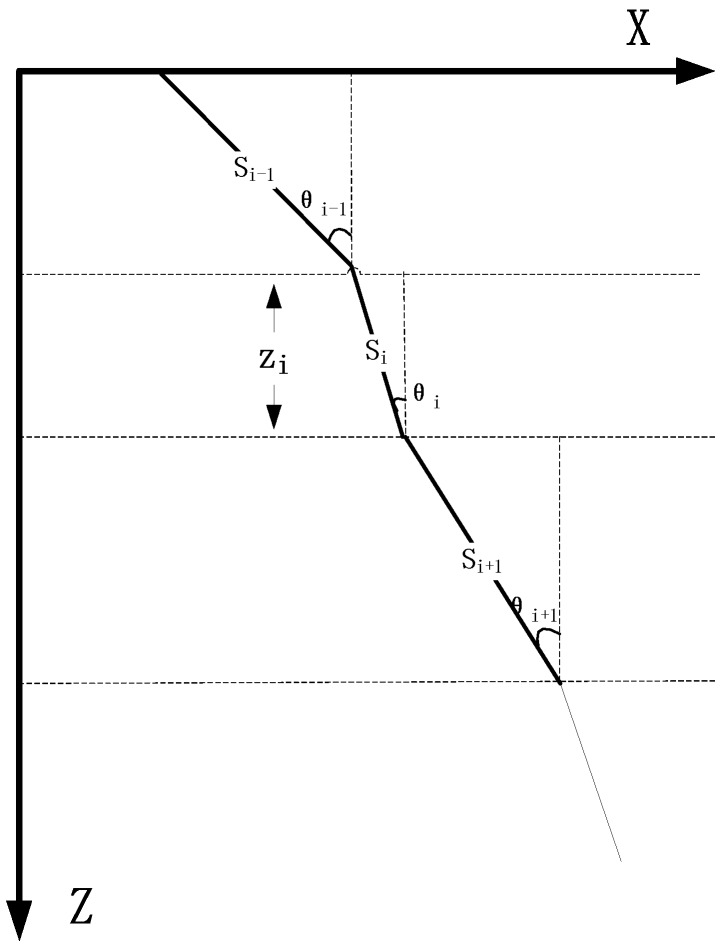
Constant sound speed ray tracing method.

Assume that the sound beam propagates in an underwater area composed of *n* layers with different sound speeds. Also, assume that a sound wave propagates along a straight line at a constant velocity in one layer, while the sound speed in each layer is different. Then, the sound wave’s propagation trajectory can be subdivided into several straight lines [27], as shown in Figure 3. The path propagates in the *i*th layer beam is $S_{i}$, the angle between the incident beam and the horizontal plane is $\theta_{i}$, $c_{i}$ is the propagation speed of a sound wave in each layer, $t_{i}$ is the wave propagation time through the *i*th layer, and $z_{i}$ is the water layer thickness of the *i*th layer.

According to the physical and geometrical functions, the trigonometric function of the acoustic wave at the incident angle $\theta_{i}$ of each medium is

(14) $$\cos\theta_{i}=\frac{z_{i}}{S_{i}}$$

The horizontal displacement $x_{i}$ and time $t_{i}$ of the acoustic wave passing through the *i*th layer are defined by

(15) $$x_{i}=z_{i}\cdot \tan\theta_{i}=\frac{p\cdot c_{i}\cdot z_{i}}{\sqrt{1-{\left(pc_{i}\right)}^{2}}}$$

(16) $$t_{i}=\frac{S_{i}}{c_{i}}=\frac{z_{i}}{\cos\theta_{i}\cdot c_{i}}=\frac{z_{i}}{c_{i}\cdot \sqrt{1-{\left(pc_{i}\right)}^{2}}}$$

The total horizontal displacement *X* of the signal received from the underwater target is defined by

(17) $$X=\sum_{i=1}^{n}x_{i}=\sum_{i=1}^{n}\frac{p\cdot c_{i}\cdot z_{i}}{\sqrt{1-{\left(pc_{i}\right)}^{2}}}$$

This method is simple and iterative. Besides, there is a small amount of calculation iterations and fast ray tracing can be achieved. However, the error made by constant sound speed ray tracing method is larger than the equal gradient ray tracing method; especially in the ocean areas where the sound speed changes greatly, so an accurate tracing cannot be achieved.

### 5.2. Equal Gradient Ray Tracing Method

**Figure 4:**
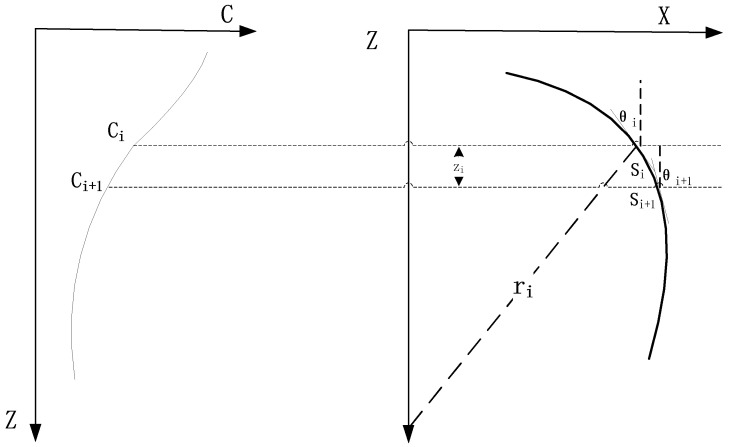
Equal gradient ray tracing method.

In the equal gradient ray tracing method, it is assumed that the relationship between the velocity of sound and the depth is linear, and the trajectory of a sound wave in each layer is considered as an arc segment with the radius r [28], as shown in Figure 4. In Figure 4, $S_{i}$ is the path of the arc segment through the *i*th layer, and $r_{i}$ is the radius of the circle corresponding to the radius of the *i*th layer; *P* is the Snell constant which is defined by Equation (13).

The sound speed gradient $g_{i}$ in the *i*th layer can be expressed as

(18) $$g_{i}=\frac{c_{i+1}-c_{i}}{z_{i}}$$

where $g_{i}$ is the sound velocity gradient of the *i*th layer.

The trajectory of a sound wave in each layer can be regarded as a circular arc with a constant curvature; the radius $r_{i}$ and the length $S_{i}$ of the arc corresponding to the *i*th layer arc can be expressed as

(19) $$r_{i}=-\frac{1}{pg_{i}}$$

(20) $$S_{i}=\frac{2\pi \cdot r_{i}}{2\pi }\left(\theta_{i+1}-\theta_{i}\right)=r_{i}\left(\theta_{i+1}-\theta_{i}\right)=\frac{\theta_{i}-\theta_{i+1}}{pg_{i}}$$

The horizontal displacement $x_{i}$ and time $t_{i}$ of the acoustic wave passing through the *i*th layer are

(21) $$x_{i}=r_{i}\left(\cos\theta_{i+1}-\cos\theta_{i}\right)=\frac{\cos\theta_{i}-\cos\theta_{i+1}}{pg_{i}}$$

(22) $$t_{i}=\int_{z_{i}}^{z_{i+1}}dz/c^{i}\left(z\right)=(1/g_{i})\ln\left(c_{i+1}/c_{i}\right)$$

The total horizontal displacement *X* of the signal received by the transmitter from the underwater target is

(23) $$X=\sum_{i=1}^{n}x_{i}=\sum_{i=1}^{n}\frac{\cos\theta_{i}-\cos\theta_{i+1}}{pg_{i}}$$

The ray path calculated by this method is in line with the actual ray path, and the calculation accuracy is higher than that of the constant sound speed ray tracing method [29]. However, the number of iterations is large, making the calculation process time-consuming.

## 6. Combined Ray Tracing Method

The USBL is used in the shallow sea environment where the sound speed varies with depth, so the sound speed can no longer be regarded as a fixed value. Using a straight sound line to track and locate the target directly will lead to inaccurate positioning. The equal gradient ray tracing method can be used to improve the positioning accuracy. However, using the equal gradient ray tracing method throughout the entire propagation, although providing high accuracy, requires a large amount of calculation of iterative processing. In the process of moving underwater targets, the deepening of the underwater depth will cause changes in the sound propagation speed. Each change needs to be recalculated, which is quite time consuming, so it is difficult to meet the demand for rapid real-time positioning of the ultra-short baselines.

**Figure 5:**
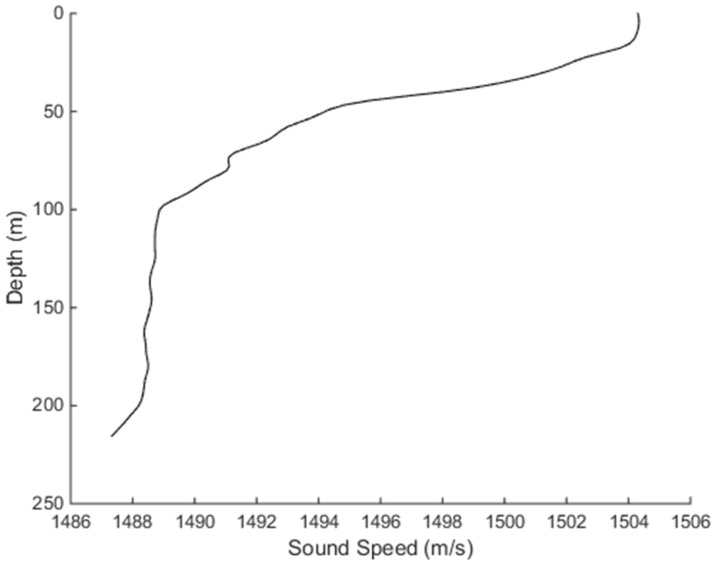
Sound speed profile dependence on the depth.

In Figure 5, the sound speed profile shows a negative gradient trend. The sound velocity is basically constant within 15 m, and the sound velocity gradient changes significantly beyond 15 m. The sound velocity tends to be stable at depth about 100 m, with a slightly negative gradient after the depth of 200 m.

In areas where the sound speed is stable, the error generated by the ray tracing is relatively small, and tracking with the constant sound speed ray tracing method still achieves good positioning accuracy. The main error in ray tracing is generated in the area where the sound speed changes greatly. In such an area, the equal gradient ray tracing method can better fit the actual situation.

In this paper, the constant sound velocity tracking method is combined with the equal gradient ray tracing method, and an improved sound ray tracing method is proposed. The principle of the proposed method is as follows. In the area where the sound speed is constant with depth, the constant sound speed tracking method is used for tracking, and in the area where the sound speed changes rapidly, the equal-gradient sound tracking method is used. By increasing the number of calculation iterations in the local area, the overall tracking accuracy can be improved.

The specific process is as follows. The gradient g of the sound speed profile between the ultra-short baseline system and the transponder is used as a judgment condition, and the gradient change threshold δ is set according to the actual situation. When g≥δ, the algorithm considers that the sound speed is greatly disturbed, so the equal gradient ray tracing method is used; when g<δ, the sound speed is considered stable, so the constant sound speed tracking method is used. The steps of the proposed method are as follows:

1. Acquire the gradient of each layer of the sound speed profile: $g_{i}$, i=1,2,⋯,n;
2. If the consecutive levels $n1_{i}^{}\;(i=1,2,\cdots ,n)$ satisfy that in each layer, $g_{i}<\delta$, then $n1_{i}^{}$ layers will be merged into one layer n1, which will be defined as a constant velocity region, and it will be considered that the sound speed value in this region is constant; accordingly, the constant sound speed ray tracing method will be used:
  (24) $${\bar{c}}_{n1}=\frac{1}{n1}\sum_{i=1}^{n1}c_{i}$$
  In Equation (24), n1 represents the number of consecutive layers in the constant-sound-speed region. Substituting the average sound speed into the constant sound speed ray tracing method, the horizontal displacement and depth of the sound wave are obtained.
3. If consecutive levels $n2_{i}^{}\;(i=1,2,\cdots ,n)$ satisfy that in each layer, $g_{i}\geq \delta$, then $n2_{i}^{}$ layers will be defined as an equal gradient sound-tracking area, and the equal gradient ray tracing method will be used in layer by layer in this area to calculate the horizontal displacement and depth of the sound wave in each layer.

**Figure 6:**
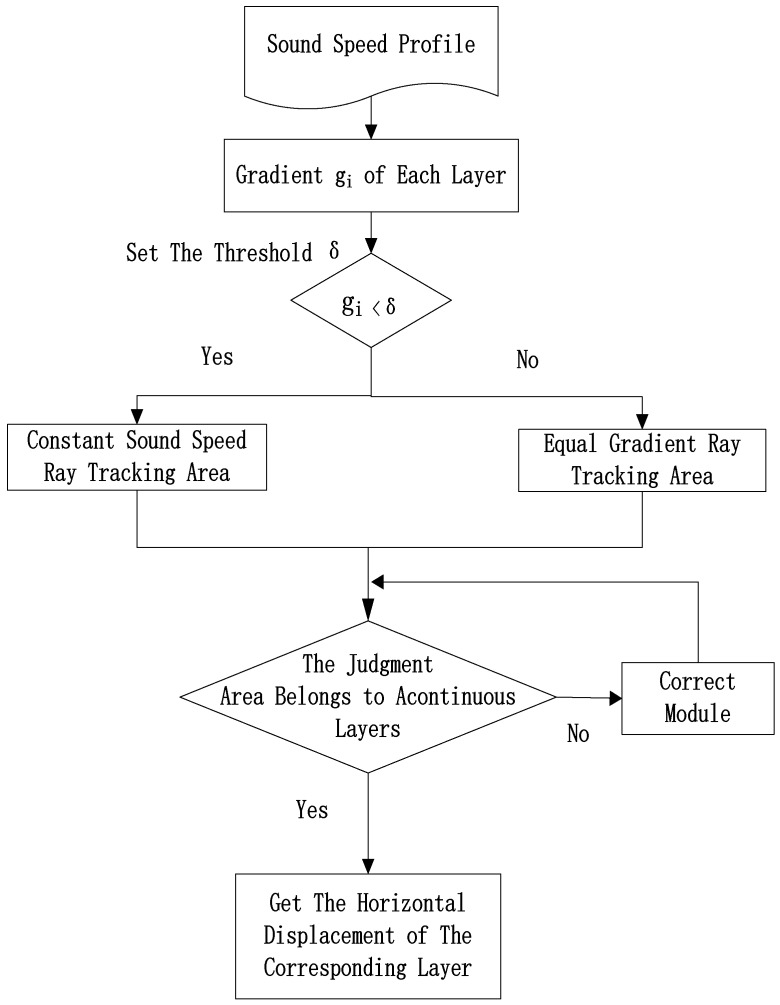
Combined ray tracing method.

The horizontal displacement of the corresponding layer is accumulated to obtain the total horizontal displacement at the corresponding depth, and after that, the slope is calculated. The above steps of the combined ray tracing method are shown by a flow chart, as shown in Figure 6.

Due to the random uncertainties in the acquisition process of the actual sound speed profile, during the implementation of the above algorithm, if there is a small number of consecutive layers in the equal gradient sound-tracking region but they satisfy $g_{i}\leq \delta$, then, it is unreasonable to use the constant sound ray tracing method. In practical applications, there are some transient sound speed stable layers in areas with the large changes in sound speed, and the intention of the proposed algorithm is to use the equal gradient ray tracing method for the whole area where the sound speed fluctuates to a greater extent. The small number of consecutive layers also need to be contained within an equal-gradient area, and the equal gradient ray tracing method is used for this area.

Therefore, the correction of an abnormal judgment is added to the algorithm. In the process of subdividing the sound speed profile, it is stipulated that if there are obvious abnormalities in the gradient of the sound level between the contiguous layers (≤3 layers), which leads to the erroneous sound tracking, then, these layers are defined as a determination abnormality. In that case, the algorithm will re-determine the abnormal layer based on the ray tracing method used in the neighboring area to achieve the corrective effect.

## 7. Traditional Sound Ray Tracing Model and Slant Distance Correction

In the USBL positioning system, the slant distance from the underwater target to the ultra-short baseline system is calculated by using the signal transmitting and receiving technique. The three-dimensional position coordinates of the underwater target are obtained by using the trigonometric functions to calculate the slant distance and azimuth angle. This work mainly describes the effect of correcting the slant distance to optimize the positioning of the USBL.

**Figure 7:**
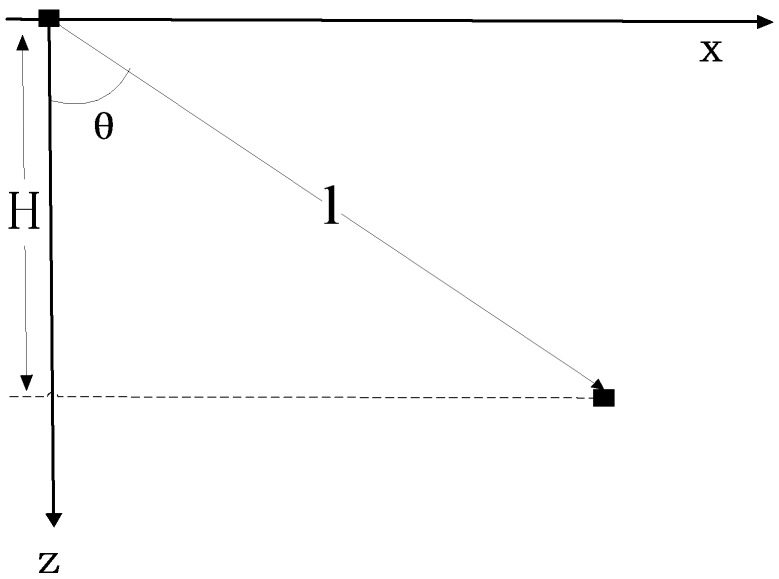
The traditional acoustic correction method.

In underwater acoustic positioning, the traditional method to find the slant distance is to use the propagation time *T* and propagation speed c of acoustic signal to obtain a direct distance l between the transponder and transmitter. The traditional method considers the sound speed is a constant value and the sound wave is set to propagate at an incident angle θ (which is also considered as the initial angle in the method below), then, it can be written that

(25) $$l=c\cdot T=\frac{H}{\cos\theta }$$

where *H* is the vertical height of the underwater target from the transmitter. The model of the traditional acoustic correction method is shown in Figure 7.

In actual situations, a sound wave does not propagate along a straight line in the shallow sea area, and its propagation path is always bent towards the direction in which the speed of sound decreases. Therefore, the ray tracing method described above can be used to approximate the actual propagation path of a sound wave using the section of a polyline or an arc. The horizontal distance *X* from transceiver head to the underwater target is obtained by summing up the layer by layer

(26) $$X=\sum_{i=1}^{n}x_{i}=\sum_{i=1}^{n}z_{i}\cdot \tan\theta_{i}$$

First, it is needed to calculate $\theta_{i}\;(i=1,2,\cdots ,n)$ using Equation (13), and the total horizontal distance *X* using Equation (26). In the USBL, the depth difference *H* between the transceiver head and the underwater target can be measured by the depth sensor, and the Pythagorean theorem can be used to obtain a more realistic slant distance between transceiver head and the underwater target $\hat{l}$, where

(27) $$\hat{l}=\sqrt{X^{2}+H^{2}}$$

## 8. Experimental Results and Analysis

In this paper, the experimental data and the result analysis are all simulation analysis. In order to make the simulation environment closer to the experimental environment, the sound velocity profile used in the simulation is the measured multiple sound speed profiles.

### 8.1. Inversion of Measured Speed Profiles

**Figure 8:**
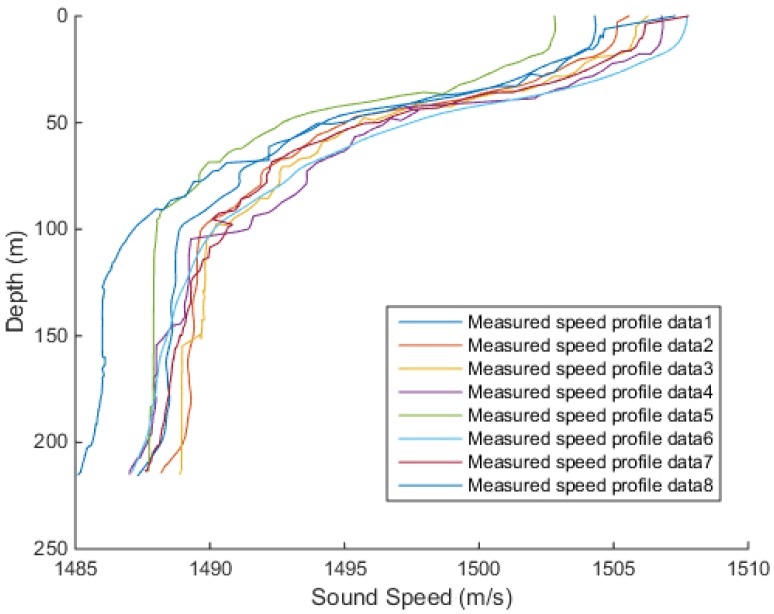
Measured speed profiles.

To verify the validity of the EOF, the measured velocity distribution samples were compared to the samples inversion by EOF. The measured speed profiles are shown in Figure 8. Due to equipment failures and environmental factors, there was an error in the measured sound speed profiles. As it can be seen in Figure 8, the sound speed fluctuated at the depth in the range 15–100 m and tended to be stable after the depth reached 100 m. The samples were measured at a maximum depth of about 200 m.

**Figure 9:**
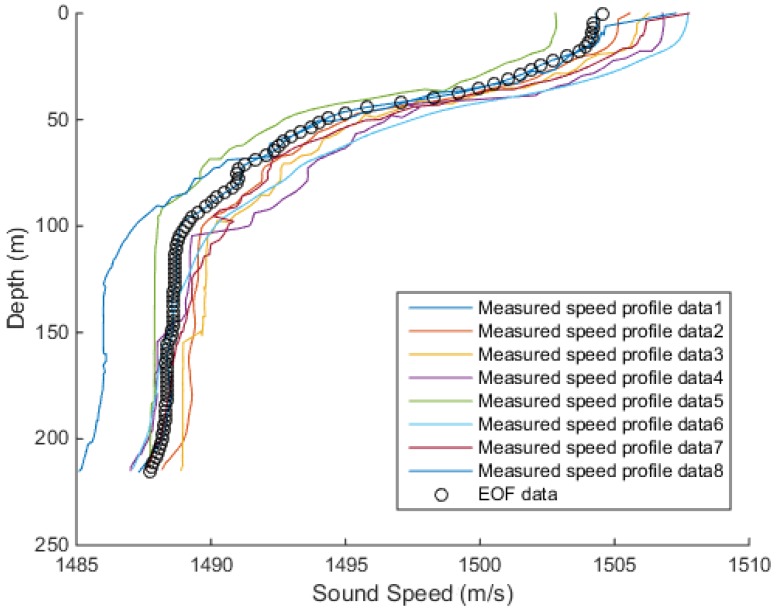
Comparison of the measured speed profiles and EOF inversion profiles.

In the simulation experiment, we performed the first three steps of the EOF. The eight sound speed profiles was extracted for fitting, and after the inversion, the newly obtained profile was compared with the measured sound speed profile, Figure 9.

**Figure 10:**
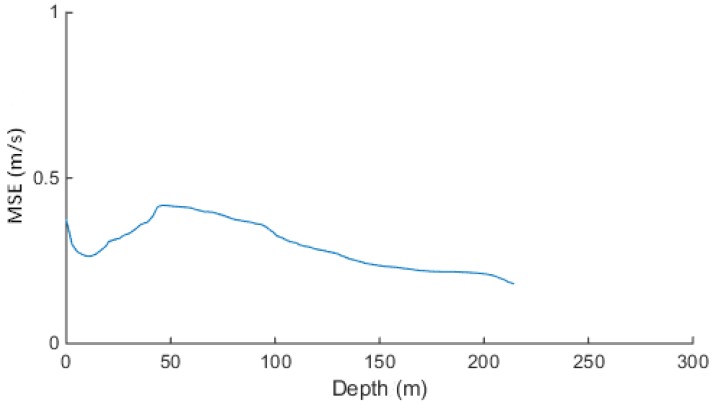
Dependence of the MSE of the sound speed profile on the depth.

The mean square error (MSE) of the sound speed profile is presented in Figure 10. In Figure 10, it can be seen that the MSE was below 0.5 m/s [30].

(28) $$\mathrm{MSE}=\frac{1}{N}{\sum_{i=1}^{N}\left({\mathrm{observed}}_{i}-{\mathrm{predicted}}_{i}\right)}^{2}$$

The MSE can evaluate the degree of change of the data. The smaller the value of the MSE, the better the accuracy of the prediction model describing the experimental data. At depths in the range 0–100 m, the MSE fluctuations were more frequent, and at depths larger than 100 m, the MSE was reduced to 0.2 m/s. Accordingly, it can be concluded that the EOF inversion of sound speed profile met the experimental requirements.

### 8.2. Experiment with Combined Ray Tracing Method

The combined ray tracing method automatically judges whether to employ the constant sound speed ray tracing method or the equal gradient ray tracing method based on the variation in sound speed gradient.

Using the EOF inversion of the sound speed profile, the depth interval between every two layers of the sound speed profile was set to be between 2 m and 3 m; thus, the sound speed profile was divided into 100 layers, and the variation in the sound speed profile gradient $g_{i}$ was obtained.

**Figure 11:**
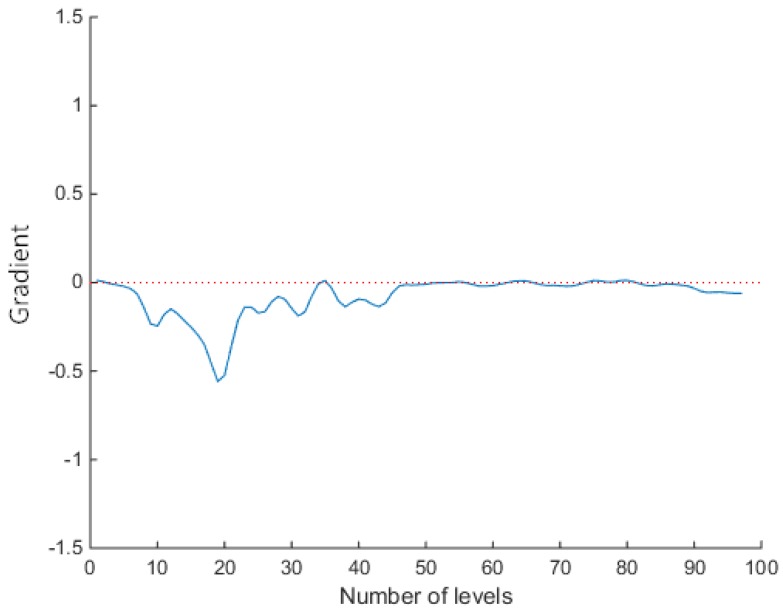
The sound speed gradient $g_{i}$.

In Figure 11, it can be seen that the gradient $g_{i}$ of the sound speed profile was stable at the 0th layer, and the sound speed was kept constant between the 0th layer and the 10th layer. Between the 10th layer and the 50th layer, $g_{i}$ significantly floated, and the sound speed changed more frequently. After the 50th layer the gradient of the sound speed $g_{i}$ tended to stabilize at zero.

We selected different speed gradient thresholds to stratify the profile. Choosing a too small or a too large threshold could cause an inadequate division of the sound speed profile, so the threshold was adjusted based on the experience and considering the actual conditions. The thresholds of 0.01, 0.06, and 0.1 were respectively used in the experiment to ensure the multiple possibilities of a regional division.

**Figure 12:**
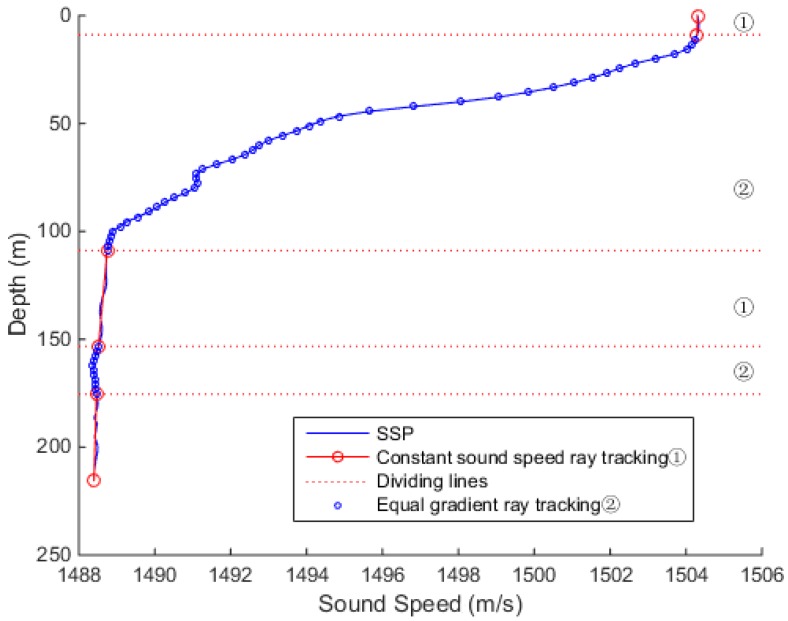
The experimental results at the threshold of 0.01.

In the case of a smaller threshold, such as that of 0.01, when $g_{i}\geq 0.01$, the sound speed profile was divided into equal-speed-gradient areas, as in Figure 12. So, the areas were interspersed with the equal gradient ray tracing method, which resulted in the unclear distinction between the divisions of the area and increased the iterations.

**Figure 13:**
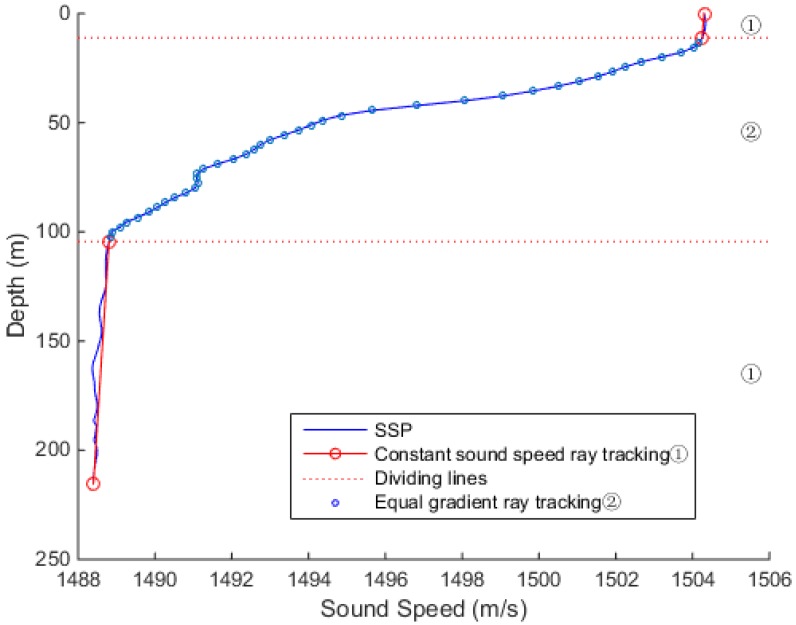
The experimental results at the threshold of 0.06.

When the threshold value was 0.06, the sound speed transform area could be distinguished well from the sound speed stable area, as shown in Figure 13. The entire sound speed profile was divided into three sections. The first section sound speed was stable, and the constant sound speed ray tracing method reduced the amount of iterations and did not produce too much error. In the second section, the equal gradient ray tracing method improved the accuracy of the complex sound speeds. In the last section, the speed of sound tended to be stable, and tracking was performed using the constant sound speed ray tracing method. By increasing the amount of calculation in the local area, the overall tracking accuracy could be improved.

**Figure 14:**
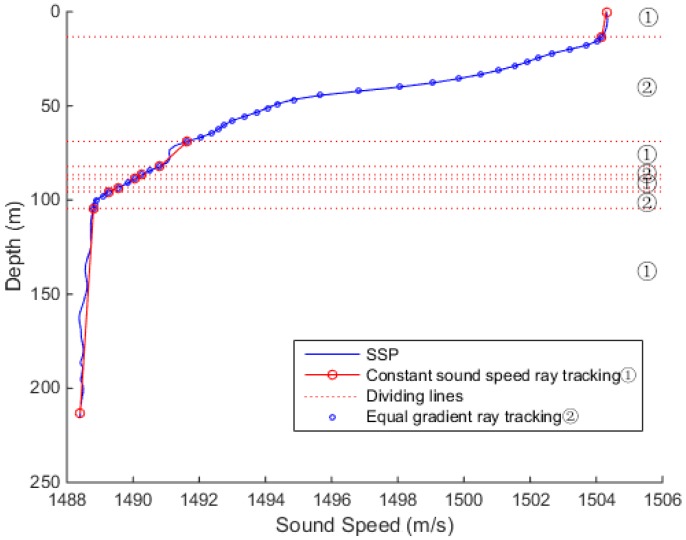
The experimental results at the threshold of 0.1.

When the threshold value was too large, such as that of 0.1 presented in Figure 14, in the region where the sound speed changed frequently, $g_{i}$ was greater than 0.01, and *G* was less than or equal to *A*, which led to the division in too many layers, and the calculation method was switched back and forth, resulting in inaccurate calculation results.

Hence, it can be concluded that when the threshold was too small or too large, the combined ray tracing method was inaccurate in judgment, and the sound speed profile was layered and disordered, resulting in errors in the sound propagation path correction. In this simulation experiment, at the threshold of 0.06, the level using constant sound speed ray tracing and the level using the equal gradient ray tracing can be better preserved, and enter the calculation of the next slant range correction.

### 8.3. Slant Distance Correction

In underwater acoustic positioning, the large azimuth angle of the sound ray will lead to the underwater sound waves diverge during the incident process, so the azimuth angle control under 70° [27,28,29,30]. The experiments here are based on the environment of the simulation experiment. In the simulation experiment, the azimuth angle and elevation angle of the USBL transmitter are both set to 30°, 40°, 45°. Therefore, we placed the target respectively at a depth of 10, 50, 100, 150, and 200 m from the USBL transmitter. For simulation purposes, we calculate the true slant range of the target at different depths by Equation (29) in simulation experiment and compare the true slant distance with the corrected slant range. We used Equation (25) for the traditional method iterations, and Equation (17) for the constant sound speed ray tracing method iterations, and compare the obtained results with the results of the proposed combined ray tracing method. The results are shown in Table 1.

(29) $$l_{true}=\sqrt{H^{2}+X^{2}}$$

where $l_{true}$ represents the slant distance of the underwater target in simulation experiment.

In Table 1, the calculation iterations parameter denotes the number of times the horizontal displacement of each layer was added due to the division of the sound speed profile layers.

**Figure 15:**
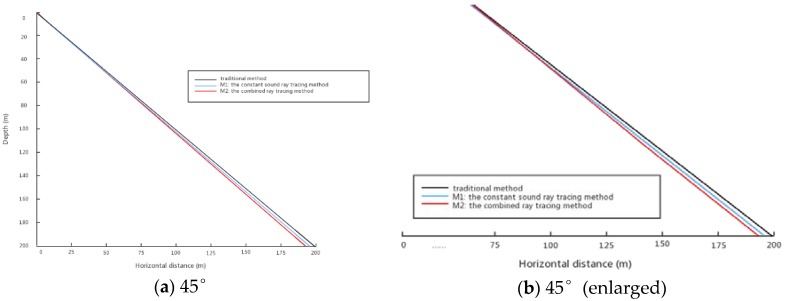
Ray tracing with an incident angle of 45 degrees.

Figure 15 shows the simulated sound ray tracing model at 45°. It can be seen that the sound ray tracing model under the traditional method is completely a straight line. At the beginning of the constant sound ray tracing method and the combined ray tracing method, the sound ray tracing almost overlaps, but gradually becomes curved later.

It can be seen from Figure 15 that in the sound line iteration, the model of sound line propagation under the traditional method has always been a straight line. However, the M1 and M2 methods have different degrees of bending.

The error analysis was used to evaluate the comparison of positioning error between different ray tracing methods. The error was defined as

(30) $$\varepsilon =\frac{\left|l_{true}-l_{new}\right|}{l_{true}}\cdot 100\%$$

where $l_{true}$ represents the true slant distance, $l_{new}$ represents the new slant distance obtained by the three methods. The comparison of the accuracy of different ray tracing methods, calculated using Equation (30), is given in Table 2, where M1 represents the constant sound ray tracing method, and M2 represents the combined ray tracing method.

As it can be seen in Table 2, the combined ray correction (method M2) exhibits an error that is always smaller than the constant sound speed ray tracing method (method M1) and using a fixed sound speed (traditional method). The worst error observed for M2 (7.4% for 40°, 10 m) is approximately one-third of the error obtained by M1 and the traditional method. 

Calculating the ratio error(M2)/error(M1), we can objectively find the reduction of the relative error when using M2 instead of M1.

From Table 3 we can see that the reduction of the relative error reduced at least 20% when using the combined ray tracing method, and the maximum reduction is about 70%. 

From the simulation results, we can find that the combined ray tracing method reduces the relative error of the slant distance in USBL without increasing the amount of calculation iterations, which has research significance and engineering application value.

## 9. Conclusions

The development of ocean needs the cooperation of many kinds of ocean equipment, various sensors are especially needed. The sensors work either independently or form an underwater sensor network [31,32,33,34,35] to accomplish the preset mission. Sufficient accurate position information is the primary prerequisite for achieving the goal.

To improve the accuracy of slant distance in the USBL positioning system, the ray tracing method is introduced in the underwater acoustic positioning system to eliminate the influence of the ray bending on the calculation of the slant distance for the positioning accuracy. This paper presents a combined ray tracing method that combines the constant sound speed ray tracing method with the equal gradient ray tracing method. The proposed method can adaptively determine which ray tracing method should be employed; namely, the constant sound speed ray tracing method is used in the regions where the sound velocity is stable, and the equal gradient ray tracing method is used in regions where the sound velocity changes significantly.

In the simulation experiments, the EOF was used to invert the real-time sound speed profile, which mitigated the errors in the measured sound velocity profile caused by the environmental and some other factors. After the sound speed profile was obtained, the combined ray tracing method autonomously judged which ray tracing method to use based on the gradient change in the sound speed profile.

The operational time of the proposed method is very short, so the sound source of the USBL remains relatively stationary with the transponder on the underwater target. To improve the positioning accuracy of the USBL positioning system further, it is also possible to optimize the sound ray correction and continuous positioning between the ultra-short baselines and moving sound sources, which is not presented in this work due to the limited space. Besides, we will conduct more in-depth studies on that subject in the future.

## Figures and Tables

**Table 1:** Slant distance and calculation iterations of various methods.

Azimuth Angle		Ray Tracing Method	True Slant Distance	Traditional Method		Constant Sound Speed Ray Tracing Method		Combined Ray Tracing Method	
	Depth (m)			Slant Distance	Calculation Iterations	Slant Distance	Calculation Iterations	Slant Distance	Calculation Iterations
30°	10		10.4	11.5	None	11.5	6	10.8	1
	50		56.4	57.7	None	57.7	24	56.7	22
	100		113.2	115.5	None	115.3	47	114.8	45
	150		170	173.2	None	172.8	69	171.9	67
	200		228.3	230.9	None	230.3	92	229.2	90
40°	10		10.8	13.1	None	13.1	6	11.6	1
	50		62.8	65.3	None	65.2	24	64.5	22
	100		125	130.5	None	130.0	47	128.4	45
	150		192.1	195.8	None	194.8	69	193.1	67
	200		256.1	261.1	None	259.6	92	257.6	90
45°	10		11.7	14.1	None	14.1	6	12.2	1
	50		65.9	70.7	None	70.6	24	67.0	22
	100		137.2	141.4	None	140.7	47	139.4	45
	150		205.2	212.1	None	210.7	69	208.4	67
	200		275.9	282.8	None	280.6	92	277.9	90

**Table 2:** Error of various ray tracing methods.

Azimuth Angle		Depth (m)	10	50	100	150	200
	Error Comparison (ε)						
	Traditional method		10.6%	2.3%	2.0%	1.9%	1.1%
30°	M1		10.6%	2.3%	1.9%	1.6%	0.9%
	M2		3.8%	0.5%	1.4%	1.1%	0.4%
	Traditional method		21.3%	4.0%	4.4%	1.9%	2.0%
40°	M1		21.3%	3.8%	4.0%	1.4%	1.4%
	M2		7.4%	2.7%	2.7%	0.5%	0.6%
	Traditional method		20.5%	7.3%	3.1%	3.4%	2.5%
45°	M1		20.5%	7.1%	2.6%	2.7%	1.7%
	M2		4.3%	1.7%	1.6%	1.6%	0.7%

**Table 3:** Ratio error(M2)/error(M1).

Ratio Error(M2)/Error(M1)	10 m	50 m	100 m	150 m	200 m
30°	36.4%	23.1%	76.2%	67.9%	45.0%
40°	34.8%	70.8%	68.0%	37.0%	42.9%
45°	20.8%	23.4%	62.9%	58.2%	42.6%

## References

[1] Han Y.F., Li Z., Zheng C.E., Sun D.J. (2015). A precision evaluation method of USBL positioning systems based on LBL triangulation. Acta Phys. Sin..

[2] Valente J.F., Alves J.C. Real-time TDOA measurements of an underwater acoustic source. Proceedings of the OCEANS 2016 MTS/IEEE Monterey.

[3] Jin L., Li J., Xu W. (2017). Tracking of time-evolving sound speed profiles with an auto-regressive state-space model. Chin. J. Acoust..

[4] Wang D.C., Guo L.H., Ding S.Q. (2004). A Scheme for High Precision Underwater Positioning. Ocean Technol..

[5] Jin S. (2006). Sound Velocity Correction and Depth Reduction in Sounding. Hydrogr. Surv. Chart..

[6] Baggeroer A.B., Kuperman W.A., Mikhalevsky P.N. (1993). An overview of matched field methods in ocean acoustics. IEEE J. Ocean. Eng..

[7] Xin M., Yang F., Yan X., BU X. (2015). An Equivalent Sound Velocity Profile Iterative Algorithm. Hydrogr. Surv. Chart..

[8] Ding J., Zhou X., Tang Q., Liu Z.C., Chen Y.L. (2004). Ray-tracking of Multibeam Echosounder System Based on Equivalent Sound Velocity Profile Method. Hydrogr. Surv. Chart..

[9] Vincent H.T., Hu S.L.J. (2002). Method and System for Determining Underwater Effective Sound Velocity. US Patent.

[10] Sun W.Q. (2007). Studies on Underwater Acoustic Localization Technique in Shallow Water and Its Application.

[11] Cheng F., Chen S., Jin S., Sun W. (2016). An Expanding Method of Sound Velocity Profiles via EOF in Marginal Deepwater Areas. Hydrogr. Surv. Chart..

[12] Yu Z. (2005). The Location Theory and Application of Across Array. Chin. J. Sci. Instrum..

[13] Shim Y., Park J., Kim J. Relative navigation with passive underwater acoustic sensing. Proceedings of the 2015 12th International Conference on Ubiquitous Robots and Ambient Intelligence (URAI).

[14] Kiran K., Najeem S., Latha G. Experimental result for direction of arrival (DOA) estimation using under water acoustic vector sensor. Proceedings of the 2015 International Symposium on Ocean Electronics (SYMPOL).

[15] Tang J.F., Yang S.E., Piao S.C. (2015). A method for calculating acoustic ray in unstratified ocean. J. Vib. Shock.

[16] Su L., Ma L., Guo S.M. (2017). Influence of Sound Speed Profile on Source Localization at Different Depths. J. Comput. Acoust..

[17] Wang X., Yang T., Wortmann M., Shi P., Hattermann F., Lobanova A., Aich V. (2017). Analysis of multi-dimensional hydrological alterations under climate change for four major river basins in different climate zones. Clim. Chang..

[18] Yang T., Cui T., Xu C.Y., Ciais P., Shi P. (2017). Development of a new IHA method for impact assessment of climate change on flow regime. Glob. Planetary Chang..

[19] Huang C., Yang T., Yeh H. (2018). Review of analytical models to stream depletion induced by pumping: Guide to model selection. J. Hydrol..

[20] Davis R.E. (1976). Predictability of Sea Surface Temperature and Sea Level Pressure Anomalies over the North Pacific Ocean. J. Phys. Oceanogr..

[21] Zhang W., Yang S., Huang Y., Lei Y. (2013). Inversion of sound speed profile in three-dimensional shallow water based on transmission time. Sci. Sin. Phys. Mech. Astron..

[22] Chen C., Ma Y., Liu Y. (2018). Reconstructing Sound speed profiles worldwide with Sea surface data. Appl. Ocean Res..

[23] Dai M., Yaan L.I. (2018). Inversion of range-dependent sound speed fields and tracking & positioning moving sound sources. J. Vib. Shock.

[24] Yan H., Wang S., Liu L., Zhou Y. (2014). A reconstruction algorithm of temperature field taking into account the bending of sound wave paths. Acta Acust..

[25] Linbang H.E., Zhao J., Zhang H., Wang X., Yan J. (2015). A precise multibeam sound ray tracing method taking into account the attitude angle. J. Harbin Eng. Univ..

[26] Xiong C., Jianjun L.I., Yan X., Liu S. (2017). Optimal Selection of Sampling Interval on Multibeam Bathymetry. Hydrogr. Surv. Chart..

[27] Zhao A.B., He W.X., Dong H.F., Hui J.Y. (2009). Positioning algorithm of deepwater USBL for random distributed velocity of sound. J. Syst. Simul..

[28] Lu X. (2012). An Improved Method for Calculating Average Sound Speed in Constant Gradient Sound Ray Tracing Technology. Geomat. Inf. Sci. Wuhan Univ..

[29] Na Q.I., Tian T. (2003). Ray tracing in multi-beam swath bathymetry. J. Harbin Eng. Univ..

[30] Wang Z., Li S., Nie Z., Wang Y., Wu S. (2016). A large incidence angle ray-tracing method for underwater acoustic positioning. Geomat. Inf. Sci. Wuhan Univ..

[31] Shen Y., Ma Y. (2000). Inversion of Sound Speed Profile for Shallow-Water Environment with Experimental Verification. J. Northwest. Polytech. Univ..

[32] Han G., Shen S., Song H., Yang T., Zhang W. (2018). A Stratification-Based Data Collection Scheme in Underwater Acoustic Sensor Networks. IEEE Trans. Veh. Technol..

[33] Han G., Jiang J., Bao N., Wan L., Guizani M. (2015). Routing Protocols for Underwater Wireless Sensor Networks. IEEE Commun. Mag..

[34] Han G., Jiang J., Sun N., Shu L. (2015). Secure Communication for Underwater Acoustic Sensor Networks. IEEE Commun. Mag..

[35] Han G., Jiang J., Shu L., Guizani M. (2015). An Attack-Resistant Trust Model based on Multidimensional trust Metrics in Underwater Acoustic Sensor Networks. IEEE Trans. Mob. Comput..

